# A transcriptome software comparison for the analyses of treatments expected to give subtle gene expression responses

**DOI:** 10.1186/s12864-022-08673-8

**Published:** 2022-06-20

**Authors:** Cung Nawl Thawng, Geoffrey Battle Smith

**Affiliations:** Biology Department and Molecular Biology Program, New Mexico State University, Las Cruces, NM USA

**Keywords:** Transcriptome software, RNA-Seq software, Small treatment differences, Low radiation, Fold-changes

## Abstract

**Background:**

In this comparative study we evaluate the performance of four software tools: DNAstar-D (DESeq2), DNAstar-E (edgeR), CLC Genomics and Partek Flow for identification of differentially expressed genes (DEGs) using a transcriptome of *E. coli.* The RNA-seq data are from the effect of below-background radiation 5.5 nGy total dose (0.2nGy/hr) on *E. coli* grown shielded from natural radiation 655 m below ground in a pre-World War II steel vault. The gene expression response to three supplemented sources of radiation designed to mimic natural background, 1952 – 5720 nGy in total dose (71–208 nGy/hr), are compared to this “radiation-deprived” treatment. In addition, RNA-seq data of *Caenorhabditis elegans* nematode from similar radiation treatments was analyzed by three of the software packages.

**Results:**

In *E. coli*, the four software programs identified one of the supplementary sources of radiation (KCl) to evoke about 5 times more transcribed genes than the minus-radiation treatment (69–114 differentially expressed genes, DEGs), and so the rest of the analyses used this KCl vs “Minus” comparison. After imposing a 30-read minimum cutoff, one of the DNAStar options shared two of the three steps (mapping, normalization, and statistic) with Partek Flow (they both used median of ratios to normalize and the DESeq2 statistical package), and these two programs identified the highest number of DEGs in common with each other (53). In contrast, when the programs used different approaches in each of the three steps, between 31 and 40 DEGs were found in common. Regarding the extent of expression differences, three of the four programs gave high fold-change results (15–178 fold), but one (DNAstar’s DESeq2) resulted in more conservative fold-changes (1.5–3.5). In a parallel study comparing three qPCR commercial validation software programs, these programs also gave variable results as to which genes were significantly regulated. Similarly, the *C. elegans* analysis showed exaggerated fold-changes in CLC and DNAstar’s edgeR while DNAstar-D was more conservative.

**Conclusions:**

Regarding the extent of expression (fold-change), and considering the subtlety of the very low level radiation treatments, in *E. coli* three of the four programs gave what we consider exaggerated fold-change results (15 – 178 fold), but one (DNAstar’s DESeq2) gave more realistic fold-changes (1.5–3.5). When RT-qPCR validation comparisons to transcriptome results were carried out, they supported the more conservative DNAstar-D’s expression results. When another model organism’s (nematode) response to these radiation differences was similarly analyzed, DNAstar-D also resulted in the most conservative expression patterns. Therefore, we would propose DESeq2 (“DNAstar-D”) as an appropriate software tool for differential gene expression studies for treatments expected to give subtle transcriptome responses.

**Supplementary Information:**

The online version contains supplementary material available at 10.1186/s12864-022-08673-8.

## Background

RNA sequencing (RNA-Seq) has a wide variety of applications in gene expression studies [1]. Since the discovery of RNA’s role as a key intermediate between the genome and the proteome, the quantification of gene expression based on the number of mRNA transcript reads is of great utility in gene expression studies [2, 3]. There are several software tools available and still in development for analysis of RNA-seq and for detection of differential expression, but there is no optimal analysis pipeline that can be used for all types of RNA-seq samples [4–7]. A successful RNA-seq study can be achieved by a good experimental design, choosing the proper type of library, the appropriate sequencing depth, and the number of replicates specific for the biological system of the study [8, 9]. The use of multiple biological replicates is critical for meaningful detection of differential gene expression [10, 11]. One challenge in comparing inter-lab results is that commercial transcriptome analyses packages have different options and parameters for transcript quantification, normalization, and differential expression analysis. In most RNA-seq experiments, the primary interest of the researcher is to find out differential gene expression between treatment and control groups. The general workflow of RNA-seq analysis includes 1. Quality control of the Next-Generation Sequencing (NGS) data (eg. adapter and low-quality trimming) 2. Mapping RNA-seq to a reference genome where the genomic information is available and 3. Quantification of the read count and detection of differentially expressed genes. In cases where a reference genome is not available, de novo transcriptome assembly is performed and differential gene expression then analyzed [5, 7, 12–14]. Successful analysis of differential gene expression depends not only on experimental design but also on the selection of appropriate software/analysis tools. Several algorithms and statistical packages have been developed for analysis of differential expression of RNA-seq including edgeR, CLC and, DESeq2 [15–18]. However, there is not much information on which software tool, and which software options, are most suitable for differential expression of RNA-seq projects.

In this study, we evaluate four commonly used RNA-seq analysis tools: DNAstar-DESeq2 (DNAstar-D), DNAstar-edgeR (DNAstar-E), CLC genomic and Partek Flow [19–21] for identification of differentially expressed genes using a RNA-Seq library of *E. coli.* The RNA-seq samples used in this study are from an on-going project on the biological effect of background and below-background radiation on organisms grown underground at the Waste Isolation Pilot Plant (WIPP) in New Mexico, USA. To compare to this radiation-shielded treatment (0.2 nGy/hr), three sources of natural radiation were supplemented to “return” cells to normal levels of background radiation. The three natural sources of radiation used were KCl (114.7 nGy/hr), and two sources of volcanic material, Tuff (207.5 nGy/hr) and Pozzolan (70.7 nGy/hr). As discussed in previous publications [22, 23], these levels in the supplemented treatments are within normal background radiation levels. Bacterial cells were exposed to these four radiation treatments for 27.5 h while incubated underground at the WIPP site. The gene expression responses to these treatments are expected to be subtle since the three supplemented radiation sources were designed to mimic the low levels that all organisms are naturally exposed to (background) and then lower levels than that (below background). Thus, this RNA-Seq data set would represent a fairly small treatment effect. In addition, we also analyzed RNA-seq from a previously published *C. elegans* nematode [23] treated with similar radiation experiments (Minus vs KCL) and compared these two model organisms responses using 3 different software. Hopefully, the analysis presented here will have application to the analyses of other subtle but biologically important gene expression projects. Our previous results have documented that mammalian cells [24], and bacteria [22, 25] exhibit a stress response to the absence of normal levels of radiation.

## Results

### Comparison of the different software pipelines used

**Fig. 1:**
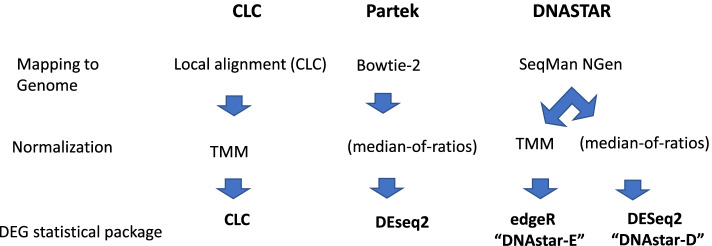
The different program mapping, normalization and statistical approaches used for each software pipeline used in this study

Figure 1 compares the different approaches used by the four software tools to map RNA reads to a genome, to normalize the data set and to statistically analyze DEGs. Each program has analytical steps shared with some of the others, for example CLC and DNAstar-E share the same normalization step (Trimmed Mean of M-values (TMM)). The statistical calculation for differential expression in the CLC software are based on General Linear Model with a negative binominal distribution, similar to EdgeR or DESeq2 (https://digitalinsights.qiagen.com/). In the Partek Flow software, several normalization and differential expression statistic packages are optionally available including DESeq2 (https://www.partek.com/partek-flow/). In the DNAstar software tool, BioConductor’s DESeq2 and edgeR are two options for statistical analysis which have their own normalization step using raw expression values of NGS to identify differentially expressed genes (https://www.dnastar.com/workflows/rna-seq/). In the CLC genomic software, the normalization method for differential gene expression is TMM. DESeq2 normalization methods use a scaling factor for a sample. DESeq2 calculates the ratio of read count to its geometric mean across all samples and the median of ratios is used for expression [16]. DESeq2 is specifically developed to find differential expressions between two conditions in studies where not many genes are differentially expressed [26]. TMM normalization method is used in DNAstar-E and CLC. After removal of the gene with the highest log expression ratio between samples, the weighted mean of log ratios between the compared sample is used as a scaling factor. TMM normalization methods assumes that most of the genes are not differentially expressed. TMM considers sample RNA populations and is effective in normalization of samples with diverse RNA repertoires [27, 28]. Neither TMM or DESeq2 normalization consider library size and gene length. CLC has an optional RPKM (Reads Per Kilobase per Million mapped reads) normalization method, but it does not provide a statistical package for analysis of differential expression studies. Similarly, Partek and DNA-star give an option for RPKM normalization, but it’s primarily used for within-sample comparisons [29]. For these reasons, we did not apply RPKM normalization for this differential gene expression study.

### Differential gene expression analysis

**Fig. 2:**
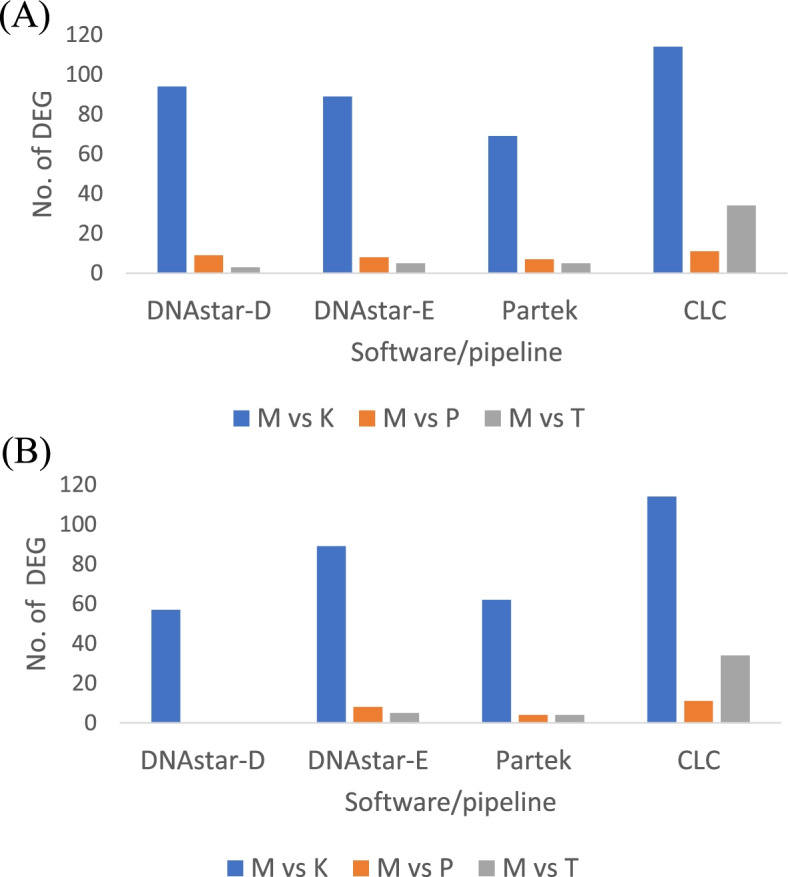
Cells were exposed to 4 treatments underground at WIPP: M = “Minus” radiation from cells grown in a steel vault, K = cells grown in a KCl Irradiator, P = cells grown in a Pozzolana irradiator and T = cells grown in a Tufo Irradiator. Detection of differentially expressed genes (DEG) with statistical significance (FDR ≤ 0.05) by four different software in *E coli*. **A** 1.5-fold cut-off  **B** 2-fold cut-off. Data includes all reads with no cut-off values

In total, 16 samples (4 replicates from each treatment) were sent for sequencing. Between 13.9 and 20.2 million raw reads of RNA-seq were obtained from each sample (Supplementary Table 1). Figure 2 shows the number of differentially expressed genes (DEGs) detected by the four software packages using a 1.5-fold cut off (A) or 2-fold cut off (B) all with statistical significance (FDR ≤ 0.05). The DEGs shown in the figure are the numbers of genes that are expressed differently in comparison to the cells grown in the absence of normal levels of radiation (the Minus, “M” treatment). The four software packages were in agreement in showing the cells grown in the KCl (“K”) irradiator had more than fivefold more DEGs compared to cells grown in the Tuff or Pozzolan irradiators (see the M vs. K comparisons in Fig. 2). As expected, the number of DEGs generally decreased at the 2-fold cutoff but the four software gave varying responses. For example, in the M vs K treatment, there was no change in number of differentially expressed genes after analyses by DNAstar-E and CLC, but the number of differentially expressed genes dropped from 94 to 54 in DNAstar-D and decreased from 69 to 62 in Partek Flow when the twofold change cutoff was applied. Similar to M vs K treatment, in M vs Pozzolan (“P”) and M vs Tuff (“T”) treatments, there were no significant genes reported by DNAstar-D while the number of DEGs remained the same in DNAstar-E and CLC. In the Partek analysis, the number of DEGs dropped from 7 to 4 in M vs P and 5 to 4 in M vs T, respectively.

### Minus radiation vs KCl-supplemented treatments

**Fig. 3:**
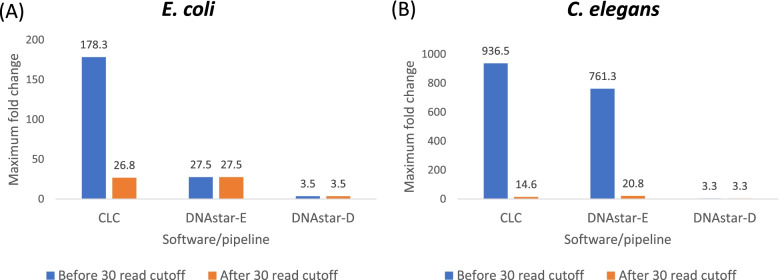
The maximum fold-change value that was detected in *E. coli* **A** and *C. elegans* **B** by each software package is shown. Only the 1.5 fold regulated genes from the “Minus” radiation (M) vs. KCl-supplemented (K) treatments are shown. Data includes all reads with no minimum read cutoff as well as after 30 read cutoff

We now will focus on M vs K treatment for further analysis throughout this paper because the number of DEGs were the highest in this comparison as reported by all four tested software/pipelines. Figure 3 shows the maximum fold change in DEGs observed in each software analysis comparing the M vs K treatments in *E. coli* (A) and *C. elegans* (B). The results demonstrated surprisingly large differences in the maximum fold-change reported by the four software packages. In *E. coli,* CLC had the highest fold-change DEG, up to 178-fold, with a total 16 genes higher than tenfold, Partek’s highest fold-change was 25.9-fold (with a total 10 genes higher than tenfold) (data not shown), DNAstar-E’s highest was 27.4-fold (a total 12 genes higher than tenfold) while the highest fold change in DNAstar-D was 3.5- fold (Fig. 3A). Similarly, in *C. elegans* analysis, the highest foldchange in CLC is 936.5-fold and 761.3-fold in DNAstar-E while 3.3-fold in DNAstar-D (Fig. 3B). When we consider the small differences in radiation dose rate among these treatments, this observation among three software programs of greater than 25-fold regulated genes caused concern regarding this potentially exaggerated response. So, we further investigated these analyses by imposing a minimum read number cutoff.

### Read count cutoff in all 8 replicates

We were concerned about the surprisingly high fold-changes of DEGs in three of the four software/pipelines, so we imposed a > 30 read count criterion on the data before analysis. Since there is not a standard read count cut-off, we examined greater than 30 read counts to make sure the data wasn’t being skewed by small read counts. So, we used only data that had greater than 30 reads in all eight replicates (4 reps from the Minus radiation and 4 from the KCl-amended treatments) in *E. coli* and four replicates (2 reps from the Minus radiation and 2 reps from the KCl-amended treatments) in *C. elegans*. (Note that since we only used the Partek trial version, we had no opportunity to change cutoff values.) The total number of DEGs produced among different software were similar in *E. coli* (61–67) but variable in *C. elegans* (74–178) after 30 read cutoff (Supplementary Fig. 1). Tables 1 and 2 shows the genes that met this criterion in the CLC and the two DNAstar sub-programs; please note that DNAstar-D and DNAstar-E have the same raw reads because they share the mapping program (SeqMan NGen) and the raw reads are shown before normalization. Table 1A also shows the fold change of the six genes that were maximally downregulated in the minus radiation M treatment compared to the KCl-supplemented treatment after we removed read counts less than 30 in all 8 replicates from *E. coli*. Table 1B shows the fold change of 13 genes after we removed reads counts less than 30 in all 4 replicates in *C. elegans*. Now, the CLC analysis resulted in maximum fold changes that were similar to the Partek and DNAstar-E pipelines. However, with these three programs showing between 10- and 26-fold downregulation, none of these programs were as conservative as the DNAstar-D pipeline which averaged twofold downregulation in *E. coli* and threefold upregulation in *C. elegans* (Table 1). After less than 30 reads have been removed, the fold change in CLC and DNAstar-E were greatly reduced but DNAstar-D was largely unaffected, being consistently conservative, and this pattern was observed with both model organisms (Fig. 3).

**Table 1:** Comparison of the highest fold change DEGs from *E. coli* (A) and *C. elegans* (B) observed in the different pipelines. Data includes only data with greater than 30 reads except for Partek which could not be adjusted

(A) Highest foldchange DEGs in *E. coli*				
**Gene Name**	**Fold change**			
	**CLC**	**Partek**	**DNAstar-E**	**DNAstar-D**
*gadA*	-26.80	-25.97	-26	-1.7
*gadB*	-22.84	-23.54	-23	-2.5
*asr*	-17.23	-17.86	-17	-1.6
*adiA*	-16.58	-17.12	-17	-1.7
*hdeB*	-10.80	-9.934	-11	-2.5
*adiC*	-10.11	NS	-10	NS
(B) Highest foldchange DEGs in *C. elegans*				
**Gene name**	**Fold Change**			
	**CLC**	**DNAstar-E**	**DNAstar-D**	
*msp-19*	NS	20.8	2.1	
*col-81*	14.6	14.4	1.6	
*col-139*	14.4	13.7	1.7	
*col-129*	13.6	13.5	1.5	
*col-133*	6.3	5.8	NS	
*fat-7*	5.7	5.3	3.3	
*asm-3*	4.9	5.0	3.0	
*K08D12.6*	3.3	3.3	NS	
*rol-8*	- 5.1	- 4.2	-1.6	
*col-17*	- 4.5	- 4.1	NS	
*sqt-2*	- 4.4	- 4.3	-1.6	
*cpt-4*	- 4.4	- 4.4	- 2.1	
*F33D4.6*	- 4.4	- 4.3	- 1.7	

*NS* detected but not statistically significant

**Table 2:** List of the number of raw reads of 8 replicates from (A) CLC, (B) DNAstar-E, (C) DNAstar-D after cutoff < 30 reads from *E. coli* . M1-4 = 4 reps from Minus radiation treatment, K1-4 = 4 reps from the KCl-amended treatment. (Note that since we only used the Partek trial version, we had no opportunity to change cutoff values.)

(A)									
**Name**	**Fold change**	**total gene reads_M1**	**total gene reads_M2**	**total gene reads_M3**	**total gene reads_M4**	**total gene reads_K1**	**total gene reads_K2**	**total gene reads_K3**	**total gene reads_K4**
*gadA*	-26	350	174	147	82	20385	2386	617	534
*gadB*	-22	265	84	68	36	9767	1439	508	333
*asr*	-17	5819	4929	909	150	2E+05	30951	11582	14226
*adiA*	-16	866	256	242	124	24125	3282	682	780
*hdeB*	-10	1223	351	568	112	16335	6740	2361	2789
*adiC*	-10	1022	241	229	68	13876	2768	888	705
(B)									
**Name**	**Fold change**	**total gene reads_M1**	**total gene reads_M2**	**total gene reads_M3**	**total gene reads_M4**	**total gene reads_K1**	**total gene reads_K2**	**total gene reads_K3**	**total gene reads_K4**
*gadA*	-26	669	323	270	144	38573	4581	1227	999
*gadB*	-23	561	201	161	94	21956	3104	1024	742
*asr*	-17	11721	9846	1774	295	356439	60822	22952	28811
*adiA*	-17	1724	496	479	246	48188	6568	1368	1569
*hdeB*	-11	2154	620	1020	186	28362	11768	4096	4844
*adiC*	-10	2044	465	452	135	27841	5547	1760	1392
(C)									
**Name**	**Fold change**	**total gene reads_M1**	**total gene reads_M2**	**total gene reads_M3**	**total gene reads_M4**	**total gene reads_K1**	**total gene reads_K2**	**total gene reads_K3**	**total gene reads_K4**
*gadA*	-1	669	323	270	144	38573	4581	1227	999
*gadB*	-2	561	201	161	94	21956	3104	1024	742
*asr*	-1	11721	9846	1774	295	356439	60822	22952	28811
*adiA*	-1	1724	496	479	246	48188	6568	1368	1569
*hdeB*	-2	2154	620	1020	186	28362	11768	4096	4844
*adiC*	NS	2044	465	452	135	27841	5547	1760	1392

**Fig. 4:**
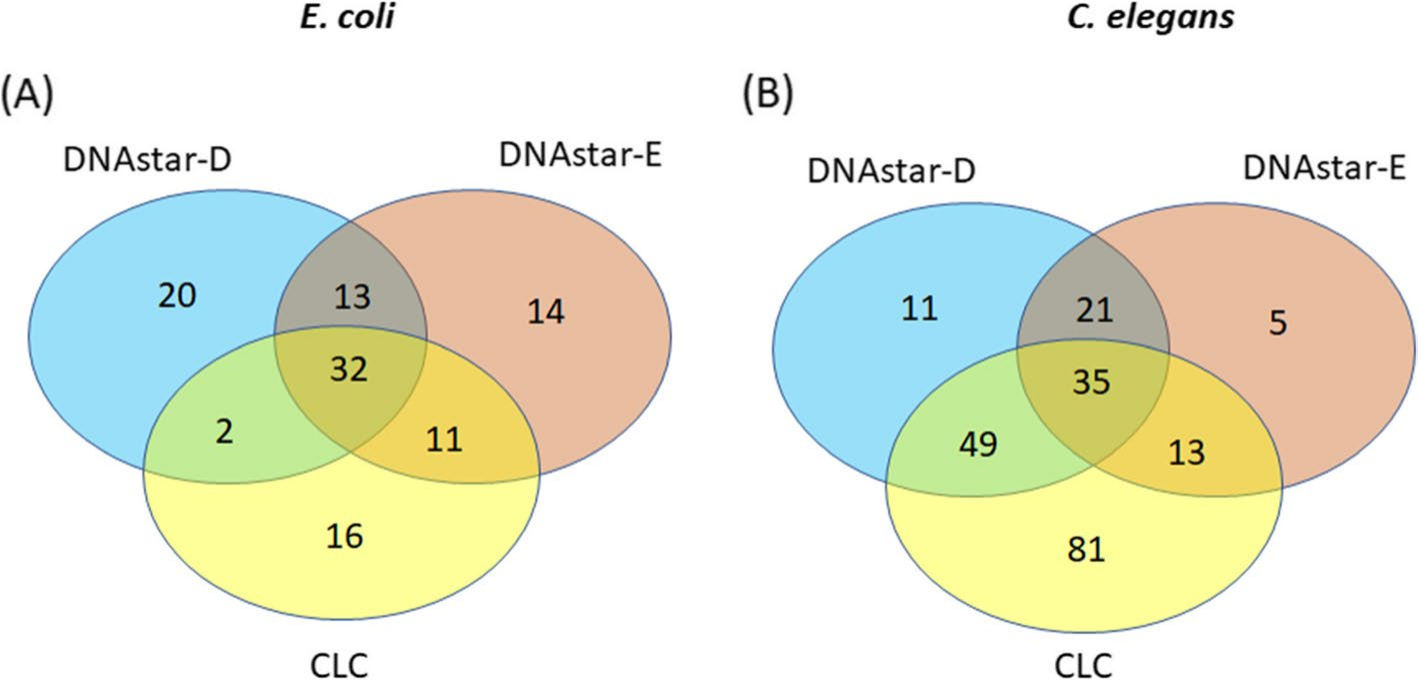
Shared DEGs comparing the “minus” radiation (M) vs KCl-supplemented (K) among three software/piplines with all reps that have more than 30 reads in *E. coli* **A** and *C. elegans* **B**

**Fig. 5:**
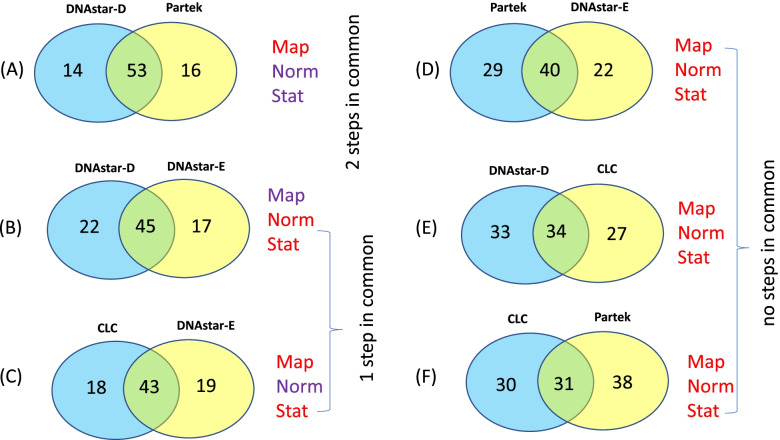
Shared DEGs of *E. coli* between different software pipelines (30 reads cutoff for all except Partek). Pipeline components that are shared are shown in black text, components that differ are shown in red

As mentioned above, we were only able to impose a 30-fold read cutoff on three of the programs, CLC, DNAStar-D and DNAStar-E. In *E. coli*, comparing CLC to the two “options” available in the DNAstar package, DNAstar-E uses the same normalization step (TMM) and they share more DEGs in common (43) than CLC vs DNAstar-D (34) which don’t share any steps in common (Fig. 4A). A total number of 32 DEGs were shared among these three softwares while 6 DEGs from DNAstar-E, 20 DEGs from DNAstar-D and 16 DEGS from CLC were unique to each package (Fig. 4A). In *C. elegans*, only 35 DEGs were commonly shared among the three software with 11 DEGs from DNAstar-D, 5 DEGs from DNAstar-E and 81 DEGs from CLC were unique to each package (Fig. 4B). The Partek and DNAstar-D programs share two of the 3 steps, that is they both use median-of-ratios to normalize and DESeq-2 to statistically analyze the data and so it makes sense that they share the most DEGs in common (53, Fig. 5A). The second highest number of shared DEGs (45) was found as a result of DNAstar-D vs DNAstar-E analyses (Fig. 5B), which shared mapping but differed in normalization and statistics. The third highest number of shared DEGs (43) was found between CLC vs DNAstar-E which shared normalization but varied in mapping and statistics (Fig. 5C). The fourth highest number of shared DEGs (40) was found in Partek and DNAstar-E where varying in mapping, normalization and statistic package between the two software (Fig. 5D). The fifth highest number of shared DEGs (34) was found in DNAstar-D vs CLC which varied mapping, normalization and DEG statistic package (Fig. 5E). The CLC and Partek programs shared the least DEGs (31) in common (Fig. 5F).

We also checked raw reads of prospective reference genes using each software package. Raw gene reads of *rrsA* in CLC were considerably lower compared to DNAstar-E(edgeR) and DNAstar-D (DESeq2), which may be the results of mapping program differences among the programs (Supplementary Table 3). To determine the high, medium, and low expression from each pipeline, we looked at the raw counts of each software package. We found that more than 400,000 gene reads in CLC was the highest expression while there was more than 900,000 total reads counts in edgeR and DEseq2 (Supplementary Tables 4, 5, 6). It appears that most of the top fold changes found in the regulated genes were within the range of medium to low expression (Table 2).

### RT-qPCR analysis

Reverse transcription PCR is the standard method to validate transcriptome gene expression patterns, and so we analyzed 22 potential *E. coli* target genes by RT-qPCR (Supplementary Table 2). The target genes were selected based on DNAStar-D’s top fold-change genes which are statistically significant (FDR adjusted *p*-value < 0.05). We analyzed the RT-qPCR differential gene expression by using three different software programs, namely CFX (BioRad), qbase + (Biogazelle) and REST (Qiagen) that normalized gene expression using the reference genes, *gyrA* and *rpoA*. Similar to the four transcriptome programs that yielded different results, the three RT-qPCR programs gave different results from the same raw data. Among 22 genes tested, the REST program showed seven genes that were significantly regulated, while the CFX program and qbase + analysis showed no genes as statistically significant even though the fold-change are very similar among the three software (Table 3). It’s worth noting that the fold change observed in the RT-qPCR analyses were most similar to the transcriptome results documented by DNAstar-D (Table 3); also note that these genes are different than the ones in Tables 1A and 2 because the genes in Table 3 were chosen to qPCR-validate the transcriptome data. For *C. elegans*, the transcriptome data have been validated with qPCR in previous studies [23].

**Table 3:** Comparison of fold change (Minus vs KCL-supplemented treatment) RT-qPCRs and Transcriptomes of *E. coli*. Please note that the genes analyzed for this validation study are based on DNAstar-D regulated genes since we found it to be the most conservative pipeline. The transcriptome data shown are ≥ 1.5 fold cutoff

Target	Fold change						
	RT-PCR			Transcriptome			
	REST	CFX	Qbase+	DNAstar-D	DNAstar-E	CLC	Partek
*sodA*	1.6	1.4	1.4	1.9	2.8	2.7	3.0
*yhiD*	-1.2	-1.3	-1.3	-2.7	-5.5	-5.5	-5.7
*zrsA*	1.1	1.0	1.0	ND	ND	ND	ND
*citF*	-1.4	-1.5	-1.5	-2.2	-8.5	-8.4	-8.6
*cysH*	-1.1	-1.2	-1.2	-3.4	-5.8	-5.7	-5.2
*hycF*	-1.0	-1.0	-1.0	-2.0	-4.5	-4.8	-4.2
*appA*	-1.2	-1.3	-1.3	-2.2	-7.5	-7.5	-7.7
*mdtF*	-1.0	-1.1	-1.1	-3.4	-6.7	-6.7	-6.9
*ydhW*	-1.2	-1.3	-1.3	-2.3	-3.8	-3.5	-3.8
*cheY*	1.4	1.5	1.3	1.6	1.6	1.5	1.6
*motA*	1.6	1.4	1.4	1.7	1.8	1.7	1.8
*napA*	1.4	1.3	1.3	2.3	9.1	9.1	8.7
*fhuA*	1.9	1.6	1.6	2.1	3.4	3.2	3.1
*fliZ*	1.7	1.5	1.5	2.1	2.4	2.4	2.4
*gadC*	1.4	1.3	1.3	-2.5	-16.9	-16.9	-16.7
*nirB*	2.5	2.2	2.2	2.1	4.8	4.6	4.4
*cyoC*	4.3	3.9	3.9	2.2	5.0	5.0	5.5
*efeO*	1.5	2.3	2.2	1.9	3.0	2.9	3.0
*hyaB*	2.0	1.7	1.7	-2.1	-7.8	-7.7	-7.5
*cysI*	-1.2	-1.3	-1.3	-2.5	-5.5	-5.7	-6.0
*slp*	-1.7	-2.0	-2.0	-2.1	-12.8	-12.8	-12.8
*mdtF*	-1.1	-1.1	-1.1	-2.5	8.0	-6.7	-6.9

gyrA and rpoA were used as reference gene

Yellow highlight = Statistically significant, *p* value ≤0.05

## Discussion

Comparative studies of analysis of transcriptome by different software tools have been reported and different softwares often give different results [6, 14, 30–33]. Researchers have to decide an appropriate software tool and pipeline based on their experimental requirements. In our study, we analyzed an *E. coli* RNA-seq using four different pipelines, DNAstar-D (DESeq2), DNAstar-E(edgeR), CLC genomic and Partek Flow. The four software programs gave contrasting results for differentially expressed genes. DNAstar-D and Partek Flow would be expected to be the most similar because they both use the same statistical package (DESeq2) and normalization programs (TMM) for DEG analysis. These two programs did have the most DEGs in common, but nevertheless identified 14 and 16 genes that were unique to each program. CLC and DNAstar-E used the same normalization step (TMM) and Partek and DNAstar-D used the same normalization (median of ratio). However, sharing normalization between the two softwares did not give similar detection of DEGs. Using different mapping programs (Partek’s Bowtie-2 vs DNAStar’s SeqMan NGen), had a strong influence on the extent of fold-regulation, averaging 18.9-fold in the case of Partek vs. 2-fold in the case of DNAStar-D.

Another important factor is the minimum number of reads used: when there was no minimum limit set, programs like CLC reported 178-fold change as its maximum, but when a 30-read-cutoff was used, this was lowered to a 25-fold maximum. Even with this more modest 25-fold maximum (which was similar to Partek and DNAstar-E software), we still did not consider this reliable considering our expected results of the small effects of these very low radiation treatments. As discussed in the introduction, the radiation levels of our supplemental sources of radiation (ranging from 71–208 nGy/hr) are near average levels of gamma (95 nGy/hr, Kendall et al. 2014) [34]. These background levels were compared to the below background treatment of 0.2 nGy/hr., and so we wouldn’t expect large fold-changes from these low level radiation treatments. Additionally, in other bacterial transcriptome studies, we’ve observed maximum fold changes of less than five [22, 35] in these reduced radiation studies. Nevertheless, these biological responses to the deprivation of natural levels of radiation, though relatively small, are consistent and statistically significant [22–25, 35].

One of the criteria to have a reliable RNA-seq analysis is having enough biological replicates [11]. In the *E. coli* study, we used four independent biological replicates from each treatment and control, but we still got different numbers of DEGs with different softwares. In our experiment with these low radiation treatments and based on our previous results [22, 24, 35], we expected small changes in gene expression patterns. Hence, we think the maximum fold change of 3.5 from the DNAstar-D pipeline is the most realistic result, in contrast to the other programs’ 20–25-fold differences in expression. Zhang et al. 2014 also observed edgeR to be less conservative (specifically it gave more false positives) than DESeq, which gave less false positives [14]. Seyednasrollah et el. 2015 reported DESeq as the safest choice because it yielded low rates of false positive with more consistent detection of DEGs than edgeR which was more variable [33]. Schaarschmidt et al. 2020 also reported that differential gene expression analysis of the data from model plant *Arabidopsis thaliana* by CLC resulted in strong divergence, with up to 50% more differentially expressed genes identified compared to DESeq2 [32]. These results are consistent with our data here reporting the number of DEGs detected in CLC (114) were twice that in DESeq2 (57) (Fig. 2 B). In order to further test these interpretations, we have re-analyzed a previously published data set incubating the *Caenorhabditis elegans* nematode under similar radiation deprived conditions [23], and we observed very similar results in terms of which pipelines gave conservative (DNAStar-D) vs exaggerated fold-changes (CLC and DNAstar-E).

Finally, we also tested RT-qPCR gene expression analysis programs to validate the transcriptome data, but two programs (CFX and qbase +) gave one result and another (REST program) differed. This is another example of different software tools giving different results from the same data. The differing results from the three RT- qPCR analyses programs likely come from the different statistical approaches used among the three softwares. The CFX and qbase + gene expression analysis is based on student *t*-tests [36], while the REST program analysis is based on an integrated randomization and bootstrapping method [37]. Nevertheless, the extent of fold-changes observed in the REST program validation analysis was most closely related to the DNAstar-D results.

## Conclusions

This study highlights the importance of examining, after DEG analysis is complete, the actual raw gene reads in order to avoid focusing on regulated genes that are statistically significant but biologically meaningless. When we excluded reads less than 30 counts, it eliminated biologically spurious data and allowed us to focus on salient results. It is difficult to say which workflow step (mapping, normalization, and statistical analysis) is the most important among the three steps because typically more than one step is varied between the programs. In the one comparison which only varied in one step (DNstar-D and Partek), mapping strongly influenced the extent of regulation as measured by fold-change. Comparing two programs at a time, DNAstar-D vs DNAstar-E and CLC vs DNAstar-E in which two steps varied, gave surprisingly very similar numbers of DEGs in common (45 and 43, respectively, Fig. 5B and C). Analyses of these 43–45 genes indicate the actual gene IDs were very similar (71–76%). Sharing statistic and normalization methods between DNAstar-D and Partek gave the highest number of DEGs but differences in mapping caused high variation in fold-changes. Sharing normalization gave only 43 DEGs, while normalization and statistics sharing gave 53 DEGs (Fig. 5A, 5C). This suggests the importance of the statistical step that influenced the higher number of DEGs.

We note the following points in summary: 1. The fold-changes identified using the DESeq2 option of DNAstar aligned with the RT-qPCR validation results. 2. This pipeline yielded more conservative results that were consistent with previous work [22, 35] and consistent with expectations that the treatments would only evoke relatively small differences in expression patterns. 3. Compared to the other software approaches tested here, DNAstar-D resulted in the expected conservative gene expression patterns in two very different model organisms (bacteria vs. nematode). And so, we consider DNAstar-D as the most conservative program for data sets in which treatment differences are expected to be small. We are hopeful these results with low-level but biologically significant expression patterns will help other researchers to choose appropriate transcriptome software pipelines as well as to distinguish between using default parameters or imposing cut-off values to more accurately analyze their data.

## Methods

### E. coli culture conditions

A minus 80℃ frozen glycerol stock of *Escherichia coli* K-12 (ATCC 10,798) was struck on TGY agar plates and incubated at 30 °C for 1 day. Four separate colonies from the agar plate were inoculated into four TGY broths (2 mL) in 15 mL tubes and incubated at 30 °C 250 rpm for 2 days. Then 20 µL of cultures were transferred to fresh TGY broth media (2 mL) and incubated overnight at 30 °C 250 rpm. After 16–18 h of incubation, the cultures were transported to WIPP and two of the biological replicates were refrigerated in a Surface lab until use the next day, and two of the reps were diluted 25 µL /10 mL of fresh TGY. The diluted cultures (1.5 mL) were transferred into the top 6 wells of four 24-well plates (MIDSCI, St. Louis, MO) and incubated at 30 °C 250 rpm. The cells incubated 24 h underground at WIPP which represented a pre-incubation before the cells were transferred again to initiate a 3.5-h incubation. Plate counts and optical densities were measured at time-zero and after 3.5 h and cells were harvested for RNA (see below). The process was repeated with the other two biological replicates of cells which had been refrigerated for 24 h. In this way, four biological replicates were carried out in this experiment. The 24-well plates were incubated underground at WIPP in four Peltier incubators (Sheldon Lab model SR13P) under the following conditions: 1. In a 15.2 cm-thick vault made from pre-World War II steel, and in 3 plastic box irradiators that surrounded cells with 14 kg of KCl, or 12.8 kg of Pozzolan or 10.8 kg of Tuff. Considering the 27.5 h exposure to the radiation treatments, the gamma doses in each of these treatments were: 0.2 nGy/hr (5.5 nGy total dose) in the vault, and 114.7 nGy/hr (3154 nGy total dose), 70.7 nGy/hr (1944 nGy total dose) and 207.5 nGy/hr (5706 nGy total dose) in the KCl, Pozzolan and Tuff irradiators. See Castillo et al. 2018 for further description of the radiation fields at WIPP.

### RNA collection and sequencing

On the 2’nd day of incubation underground, exponential phase (3.5 h) cultures of *E. coli* was harvested as follows: 1 mL of RNA protect solution was added into 0.5 mL of culture and kept at room temperature for 5 min after mixing well. Cell pellets were harvested by centrifugation at 12,000 rpm for 5 min. The supernatant was decanted, and the pellet was kept at -20 °C. RNA was extracted from the cell pellet using an RNA isolation kit (RNeasy@ Mini Kit, QIAGEN) according to manufacturer’s instruction. The quantity and quality of RNA was evaluated by Nano drop and by running on agarose gel electrophoresis. The total RNA samples were sent for sequencing at Novogene (Sacramento, CA). For *E. coli* library construction, rRNA was removed using the Ribo-Zero kit that leaves mRNA. First, mRNA was fragmented randomly by adding fragmentation buffer, then the cDNA was synthesized by using mRNA template and random hexamers primer, after which a custom second-strand synthesis buffer (Illumina), dNTPs (dUTP, dATP, dGTP and dCTP), RNase H and DNA polymerase I were added to initiate the second-strand synthesis. This was followed by purification by AMPure XP beads, terminal repair, polyadenylation (for bacteria), sequencing adapter ligation, size selection and degradation of second-strand U-Contained cDNA by the USER enzyme. The strand-specific cDNA library was generated after the final PCR enrichment. Library concentration was first quantified using a Qubit 2.0 fluorometer (Life Technologies), and then diluted to 1 ug/µl before checking insert size on an Agilent 2100 and quantifying to greater accuracy by quantitative PCR (Q-PCR) (Library activity > 2 Nm). Qualified libraries were sequenced on an Illumina Nova Seq 6000 Platform (Illumina, San Diego, CA, USA) using a paired-end 150 run (2 × 150 bases).

### Data analysis

For the transcriptome analyses, CLC Genomic Workbench 12.2 (Qiagen Bioinformatics, Germantown, MD, USA) was used. All RNA Seq data were screened for False Discovery Rate (FDR), and were accepted if FDR < 0.05 [38]. Raw RNA sequences were trimmed, aligned, and mapped against the reference genome of *E. coli* K-12 MG1655 (NC_000913.3) in CLC program with the following parameters: 2 maximum mismatches, 90% minimum similarity fraction, and 10 maximum hits per read for mapping [39]. The raw RNA-seq were also analyzed by DNAStar (Madison, Wisconsin, USA) with SeqMan N Gen (version 17.2.1.61) and Partek Flow (Partek Inc., St. Louis, MO, USA). Other than using a 30-base read cutoff as indicated, default parameters of each software tool was used for all RNA-seq analyses. A summary of the different software pipelines is shown in Fig. 1. The raw RNA sequences obtained in this study were deposited at NCBI database (Accession number PRJNA787903). We also obtained RNA-seq (NCBI accession no. PRJNA631208) from our previous studies about *C. elegans* nematode response to low background radiation [23]. The experiments were conducted in two biological replicates for each treatment (Minus) as well as control (KCL). The raw RNA-seq were analyzed based on the materials and methods above using three programs: CLC, DNAstar-E (edgeR) and DNAstar-D (DESeq2). We don’t have access to Partek as we used only trial version.

### RT-qPCR

The validity of differential expression was verified by using RT-qPCR for direct comparison with RNA Seq. The qPCR reactions (10 uL) were performed in triplicate using iTaq Universal One-Step RT-qPCR kit (BioRad, Hercules, CA, USA) with 0.5 μM of each primer (Supplementary Table 2), and 1 ng of total RNA as template. First cDNA was synthesized by reverse transcription at 50 °C for 10 min followed by reverse transcriptase inactivation at 95 °C for 1 min. The reaction was directly followed by PCR amplification as follows: 40 cycles of denaturation: 30 s at 95 °C; annealing: 30 s at 55 °C; and extension: 30 s at 72 °C. The final PCR step was 30 s at 96 °C followed by 5 s at 60 °C and the PCR reaction was stopped by a constant temperature of 4 ℃. The relative expression of the target genes *was* calculated using *gyrA and rpoA* as reference genes and using the efficiency-corrected REST model [37], CFX Maestro software version 2.2 (BioRad) and qbase + version 3.3 [36]. The *gyr*A and *rpoA* genes were chosen using reference gene selection tool CFX Maestro software version 2.2 (BioRad). For each comparison, four biological replicates and three technical replicates were used for all calculations.

## Supplementary Information

**Additional file 1:** **SupplementaryTable 1.** The raw reads obtained from RNA-seq of *E. coli* in thisstudy. **Supplementary Table 2. **Primers used in this study for qPCR. **Supplementary Table 3. **Comparison of housekeeping gene raw counts from different software. **SupplementaryTable 4.** High, medium and low expression genes from CLC genomic. **SupplementaryTable 5.** High, medium and low expression from DNAstar( EdgeR). **SupplementaryTable 6.** High, medium and low expression from DNAstar( DESeq2). **Supplementary Figure1.** The number of differentially expressed genes (DEGs) in *E. coli* **A** and *C. elegans* **B** before and after 30 read cutoff.

## Data Availability

All data generated or analyzed during this study are included in this published article and its supplementary information files. The raw RNA sequences obtained in this study were deposited at NCBI database (Accession number PRJNA787903).

## Abbreviations

WIPP: Waste Isolation Pilot Plant
DEG: Differentially expressed genes
NGS: Next-Generation Sequencing
