# Supplementary material for: A transcriptome software comparison for the analyses of treatments expected to give subtle gene expression responses
Source: BMC Genomics. 2022 Jun 20;23:452. doi: 10.1186/s12864-022-08673-8 (PMC9208185; doi:10.1186/s12864-022-08673-8)
Supplement: Supplementary file 1 — Additional file 1: SupplementaryTable 1. The raw reads obtained from RNA-seq of E. coli in thisstudy. Supplementary Table 2. Primers used in this study for qPCR. Supplementary Table 3. Comparison of housekeeping gene raw counts from different software. SupplementaryTable 4. High, medium and low expression genes from CLC genomic. SupplementaryTable 5. High, medium and low expression from DNAstar( EdgeR). SupplementaryTable 6. High, medium and low expression from DNAstar( DESeq2). Supplementary Figure1. The number of differentially expressed genes (DEGs) in E. coli A and C. elegans B before and after 30 read cutoff. [file 12864_2022_8673_MOESM1_ESM.docx]

Supplementary Table 1. The raw reads obtained from RNA-seq of *E. coli* in this study

| **Sample** | **Raw reads** | **Raw data(G)** | **Effective(%)** | **Error(%)** | **Q20(%)** | **Q30(%)** | **GC(%)** |
| --- | --- | --- | --- | --- | --- | --- | --- |
| M1 | 14,686,726 | 2.2E+09 | 99.33 | 0.02 | 98.17 | 94.58 | 51.35 |
| M2 | 13,874,738 | 2.08E+09 | 99.56 | 0.02 | 98.13 | 94.47 | 51.37 |
| M3 | 13,914,958 | 2.09E+09 | 99.12 | 0.02 | 98.09 | 94.43 | 51.46 |
| M4 | 17,809,978 | 2.67E+09 | 99.05 | 0.02 | 98.25 | 94.79 | 51.88 |
| K1 | 15,225,034 | 2.28E+09 | 99.56 | 0.02 | 98 | 94.22 | 51.32 |
| K2 | 16,791,176 | 2.52E+09 | 99.58 | 0.02 | 98.29 | 94.82 | 51.41 |
| K3 | 14,804,130 | 2.22E+09 | 99.38 | 0.02 | 98.17 | 94.61 | 51.36 |
| K4 | 16,414,008 | 2.46E+09 | 99.38 | 0.02 | 98.06 | 94.35 | 51.4 |
| P1 | 14,863,084 | 2.23E+09 | 99.53 | 0.02 | 98.21 | 94.66 | 51.37 |
| P2 | 18,167,882 | 2.73E+09 | 99.57 | 0.02 | 98.15 | 94.47 | 51.37 |
| P3 | 17,824,870 | 2.67E+09 | 99.08 | 0.02 | 98.19 | 94.58 | 51.35 |
| P4 | 14,693,338 | 2.2E+09 | 99.52 | 0.02 | 98.05 | 94.3 | 51.43 |
| T1 | 16,153,010 | 2.42E+09 | 99.65 | 0.02 | 98.18 | 94.6 | 51.45 |
| T2 | 20,191,234 | 3.03E+09 | 99.59 | 0.02 | 98.19 | 94.63 | 51.41 |
| T3 | 17,142,984 | 2.57E+09 | 99.4 | 0.02 | 98.19 | 94.62 | 51.3 |
| T4 | 14,870,930 | 2.23E+09 | 99.52 | 0.02 | 98.18 | 94.62 | 51.41 |

Supplementary Table 2. Primers used in this study for qPCR.

| **Gene** | **Function** | **Sequence (5' to 3')** | | **Size (bp)** |
| --- | --- | --- | --- | --- |
|  |  | **Forward** | **Reverse** |  |
| *fliz* | DNA-binding transcriptional regulator FliZ | GCGCAAACGAACGACATATT | ATGAAAATGGCTGGCAAACG | 176 |
| *cheY* | chemotaxis protein CheY | GCCTGCAACTTATTGAGAGC | GATGACTTTTCCACCATGCG | 110 |
| *motA* | motility protein A | CGTGCGTCTCAATCTCTTCA | GATGTTTTCGCTGGAACGTG | 185 |
| *napA* | periplasmic nitrate reductase subunit NapA | GACCAAACCATGCGTACTCT | TACGAAGTGCTGTATGCCAC | 139 |
| *nirB* | nitrite reductase catalytic subunit NirB | GGACTGCTTTGTCTATCGCA | CCATCAGCATAGGGGCAAAT | 173 |
| *cyoC* | cytochrome bo3 ubiquinol oxidase subunit 3 | GGTGATGGAGCTGAACAACA | GCGACTGCATTCTGTTCTCT | 143 |
| *fhuA* | ferrichrome outer membrane transporter/phage receptor | ACCGGCACTAAAACCGATAC | TCTTTTACCGACTTCGGCTG | 101 |
| *efeO* | ferrous iron transport system protein EfeO | GATCTGGATGGCAGCATTGA | TAAAGCTGCTCAGCGTACTG | 146 |
| *sodA* | Superoxide dismutase | CGGCTCCGTTGATAACTTCA | AATAGCTTCACCCATCAGCG | 148 |
| *zraS* | sensory histidine kinase ZraS | TATTCAGGCGATTGGTCAGC | AGTGGTGAAGTACGGAGTGA | 142 |
| *gadC* | L-glutamate:4-aminobutyrate antiporter | CAATTGCCACGACCAGTTTC | AACATGTCCTTCCTGATCGC | 163 |
| *yhiD* | inner membrane protein YhiD | CTTCTATCGACGTTGTGGCA | ACGGTATCAATGCTCGACTG | 127 |
| *hycF* | formate hydrogenlyase subunit HycF | ATTCCGGGCAAGTCTCAAAG | GCGGTGTGGAAGAAAGAAGA | 178 |
| *citF* | citrate lyase alpha subunit | ATAAGGCAGCAGTTCTTCGG | CGTCCTGTGATGAATTCGGT | 131 |
| *hyaB* | hydrogenase 1 large subunit | GCTTATCACAAAGGCGATGC | ATACTGCAACTTACCTGCGG | 150 |
| *appA* | periplasmic phosphoanhydride phosphatase | CTGAAGCTGGAAAGTGTGGT | TCAGCCAACCCAGTTTTACC | 127 |
| *cysH* | phosphoadenosine phosphosulfate reductase | GGTTCCCATTTACGGGTTGT | TCGACTGGGATAACCGAACT | 121 |
| *hdeB* | periplasmic acid stress chaperone | AGTAACGGTATCGCCACCT | TCACTGGTGAACGCACAATC | 156 |
| *mdtF* | multidrug efflux pump RND permease MdtF | TATGATGCTTGCAGGTGGTC | CAATCACCTGAGTGACCGAG | 143 |
| *ydhW* | Redox enzyme maturation protein | AATCATCCTTGCTTCCCGTC | GCTGAAATCCTGGCTGAGTT | 181 |
| *gyrA ^a^* | DNA gyrase subunit A | TCAGCGGAGAACAGCATTAC | TGACCGAGTTTGAAGAAGGC | 190 |
| *^rpoA a^* | RNA polymerase subunit alpha | CCGCGCTGAACTTTGATACG | CTGCTCAACCTGAAAGGGCT | 203 |
| *napH* | ferredoxin-type protein NapH | TTATCACCGTGCTCTACGCC | CCAACAGCACGTAGCGAATG | 156 |
| *cysI* | sulfite reductase, hemoprotein subunit | ATGAAGAACCGATCCTCGGC | GTTAAAGCCCACCAGCTTGC | 146 |
| *slp* | starvation lipoprotein | GCGAAGCCTGATATTGAAGCC | ATGGTGCCGAGGATGGTAAC | 110 |
| *mdtE* | multidrug efflux pump membrane fusion protein MdtE | ACAACGTCTGGACCCGATTT | GTCGGAGAATTTCAGCGTGC | 175 |

*^a^* Reference genes used for gene expression normalization.

Supplementary Table 3. Comparison of housekeeping gene raw counts from different software.

* As expected for reference genes, fold changes values were not statistically significant (FDR > 0.05)

1. Raw counts from CLC genomic

|  | **fold change** | **M1** | **M2** | **M3** | **M4** | **K1** | **K2** | **K3** | **K4** |
| --- | --- | --- | --- | --- | --- | --- | --- | --- | --- |
| ***gyrA*** | 2.1 | 2425 | 2499 | 2553 | 12763 | 2164 | 2693 | 2227 | 2671 |
| ***gyrB*** | 1.1 | 4111 | 4689 | 4608 | 7887 | 4049 | 5449 | 4690 | 5420 |
| ***rpoA*** | 1.5 | 53110 | 67828 | 58328 | 147502 | 31607 | 57895 | 66139 | 58183 |
| ***rrsA*** | 1.8 | 7 | 0 | 4 | 10 | 10 | 0 | 0 | 12 |

1. Raw counts from DNAstar (DESEq2)

|  | **fold change** | **M1** | **M2** | **M3** | **M4** | **k1** | **K2** | **K3** | **K4** |
| --- | --- | --- | --- | --- | --- | --- | --- | --- | --- |
| ***gyrA*** | 1.5 | 4905 | 5026 | 5134 | 25930 | 4333 | 5383 | 4481 | 5357 |
| ***gyrB*** | 1.1 | 8216 | 9337 | 9206 | 15743 | 8118 | 10853 | 9364 | 10789 |
| ***rpoA*** | 1.3 | 99482 | 126362 | 109780 | 281277 | 58925 | 107931 | 123372 | 108724 |
| ***rrsA*** | 1.2 | 3296 | 3055 | 2998 | 9770 | 2962 | 3309 | 3486 | 4144 |

1. Raw counts from DNAstar (EdgeR)

|  | **fold change** | **M1** | **M2** | **M3** | **M4** | **k1** | **K2** | **K3** | **K4** |
| --- | --- | --- | --- | --- | --- | --- | --- | --- | --- |
| ***gyrA*** | 2.0 | 4905 | 5026 | 5134 | 25930 | 4333 | 5383 | 4481 | 5357 |
| ***gyrB*** | 1.2 | 8216 | 9337 | 9206 | 15743 | 8118 | 10853 | 9364 | 10789 |
| ***rpoA*** | 1.6 | 99482 | 126362 | 109780 | 281277 | 58925 | 107931 | 123372 | 108724 |
| ***rrsA*** | 1.3 | 3296 | 3055 | 2998 | 9770 | 2962 | 3309 | 3486 | 4144 |

Supplementary Table 4. High, medium and low expression genes from CLC genomic

A. CLC_ high expression (100,000 – 400,000 reads)

| **Name** | **biotype** | **Total gene reads_M1** | **Total gene reads_M2** | **Total gene reads_M3** | **Total gene reads_M4** | **Total gene reads_K1** | **Total gene reads_K2** | **Total gene reads_K3** | **Total gene reads_K4** |
| --- | --- | --- | --- | --- | --- | --- | --- | --- | --- |
| *ssrA* | ncRNA | 320488 | 238684 | 309348 | 83149 | 300,141 | 273,639 | 269,286 | 469116 |
| *pflb* | Protein coding | 136461 | 77348 | 111295 | 49177 | 254,321 | 225,490 | 125,166 | 146412 |
| *ompC* | Protein coding | 151590 | 156414 | 118604 | 80903 | 194,776 | 212,156 | 158,434 | 162899 |

B. CLC_ medium expression (5000-50,000 reads)

| **Name** | **biotype** | **Total gene reads_M1** | **Total gene reads_M2** | **Total gene reads_M3** | **Total gene reads_M4** | **Total gene reads_K1** | **Total gene reads_K2** | **Total gene reads_K3** | **Total gene reads_K4** |
| --- | --- | --- | --- | --- | --- | --- | --- | --- | --- |
| *dapA* | Protein coding | 8247 | 6854 | 6599 | 7662 | 10,932 | 10,090 | 6738 | 7694 |
| *rpsG* | Protein coding | 16459 | 21427 | 20341 | 30320 | 10.852 | 22.301 | 23416 | 22555 |
| *hslU* | Protein coding | 6867 | 2635 | 5311 | 7785 | 10,465 | 10,459 | 6108 | 7807 |

C. CLC_ low expression (50 – 500 reads)

| **Name** | **biotype** | **Total gene reads_M1** | **Total gene reads_M2** | **Total gene reads_M3** | **Total gene reads_M4** | **Total gene reads_K1** | **Total gene reads_K2** | **Total gene reads_K3** | **Total gene reads_K4** |
| --- | --- | --- | --- | --- | --- | --- | --- | --- | --- |
| *xdhA* | Protein coding | 154 | 88 | 48 | 42 | 101 | 141 | 91 | 130 |
| *uspC* | Protein coding | 73 | 49 | nd | nd | 100 | 49 | nd | 50 |
| *yeis* | Protein coding | 102 | 68 | 68 | 164 | 100 | 76 | 72 | 104 |

Supplementary Table 5. High, medium and low expression from DNAstar( EdgeR).

A. DNAstar-E(edgeR)_ high expression

| **Name** | **Total reads count_M1** | **Total reads count_M2** | **Total reads count_M3** | **Total reads count_M4** | **Total reads count_K1** | **Total reads count_K2** | **Total reads count_K3** | **Total reads count_K4** |
| --- | --- | --- | --- | --- | --- | --- | --- | --- |
| ssrA | 643404 | 479468 | 621715 | 168634 | 603843 | 549947 | 540628 | 941974 |
| pflb | 267533 | 151218 | 219293 | 98447 | 498524 | 442913 | 246153 | 287361 |
| ompC | 286532 | 296448 | 226080 | 162222 | 370158 | 401370 | 300868 | 309684 |

B. DNAstar-E(edgeR)_ medium expression

| **Name** | **Total reads count_M1** | **Total reads count_M2** | **Total reads count_M3** | **Total reads count_M4** | **Total reads count_K1** | **Total reads count_K2** | **Total reads count_K3** | **Total reads count_K4** |
| --- | --- | --- | --- | --- | --- | --- | --- | --- |
| dapA | 16297 | 13485 | 13138 | 15344 | 21495 | 19861 | 13355 | 15236 |
| rpsG | 27379 | 35285 | 34234 | 54895 | 17716 | 36621 | 38923 | 37290 |
| hslU | 13577 | 12343 | 10548 | 15724 | 20703 | 20690 | 12084 | 15371 |

C. DNAstar-E(edgeR)_ low expression

| **Name** | **Total reads count_M1** | **Total reads count_M2** | **Total reads count_M3** | **Total reads count_M4** | **Total reads count_K1** | **Total reads count_K2** | **Total reads count_K3** | **Total reads count_K4** |
| --- | --- | --- | --- | --- | --- | --- | --- | --- |
| xdhA | 285 | 162 | 99 | 83 | 201 | 274 | 175 | 252 |
| uspC | 145 | 95 | 20 | 14 | 199 | 96 | 23 | 103 |
| yeis | 229 | 147 | 143 | 353 | 199 | 186 | 139 | 186 |

Supplementary Table 6. High, medium and low expression from DNAstar( DESeq2)

A. DNAstar-D(Deseq2)_ high expression

| **Name** | **Total reads count_M1** | **Total reads count_M2** | **Total reads count_M3** | **Total reads count_M4** | **Total reads count_K1** | **Total reads count_K2** | **Total reads count_K3** | **Total reads count_K4** |
| --- | --- | --- | --- | --- | --- | --- | --- | --- |
| ssrA | 643404 | 479468 | 621715 | 168634 | 603843 | 549947 | 540628 | 941974 |
| pflb | 267533 | 151218 | 219293 | 98447 | 498524 | 442913 | 246153 | 287361 |
| ompC | 286532 | 296448 | 226080 | 162222 | 370158 | 401370 | 300868 | 309684 |

B. DNAstar-D(Deseq2)_ medium expression

| **Name** | **Total reads count_M1** | **Total reads count_M2** | **Total reads count_M3** | **Total reads count_M4** | **Total reads count_K1** | **Total reads count_K2** | **Total reads count_K3** | **Total reads count_K4** |
| --- | --- | --- | --- | --- | --- | --- | --- | --- |
| dapA | 16297 | 13485 | 13138 | 15344 | 21495 | 19861 | 13355 | 15236 |
| rpsG | 17716 | 36621 | 38923 | 37290 | 27379 | 35285 | 34234 | 54895 |
| hslU | 20703 | 20690 | 12084 | 15371 | 13577 | 12343 | 10548 | 15724 |

C. DNAstar-D(Deseq2)_ low expression

| **Name** | **Total reads count_M1** | **Total reads count_M2** | **Total reads count_M3** | **Total reads count_M4** | **Total reads count_K1** | **Total reads count_K2** | **Total reads count_K3** | **Total reads count_K4** |
| --- | --- | --- | --- | --- | --- | --- | --- | --- |
| xdhA | 201 | 274 | 175 | 252 | 285 | 162 | 99 | 83 |
| uspC | 199 | 96 | 23 | 103 | 145 | 95 | 20 | 14 |
| yeis | 199 | 186 | 139 | 186 | 229 | 147 | 143 | 353 |

Supplementary Figure 1. The number of differentially expressed genes (DEGs) in *E. coli* (A) and *C. elegans* (B) before and after 30 read cutoff.
